# Using Electronic Health Record–Based Clinical Decision Support to Provide Social Risk–Informed Care in Community Health Centers: Protocol for the Design and Assessment of a Clinical Decision Support Tool

**DOI:** 10.2196/31733

**Published:** 2021-10-08

**Authors:** Rachel Gold, Christina Sheppler, Danielle Hessler, Arwen Bunce, Erika Cottrell, Nadia Yosuf, Maura Pisciotta, Rose Gunn, Michael Leo, Laura Gottlieb

**Affiliations:** 1 Kaiser Permanente Center for Health Research Portland, OR United States; 2 OCHIN, Inc. Portland, OR United States; 3 University of California San Francisco San Francisco, CA United States

**Keywords:** social determinants of health, decision support systems, clinical, electronic health records, community health centers, health status disparities

## Abstract

**Background:**

Consistent and compelling evidence demonstrates that social and economic adversity has an impact on health outcomes. In response, many health care professional organizations recommend screening patients for experiences of social and economic adversity or *social risks*—for example, food, housing, and transportation insecurity—in the context of care. Guidance on how health care providers can act on documented social risk data to improve health outcomes is nascent. A strategy recommended by the National Academy of Medicine involves using social risk data to adapt care plans in ways that accommodate patients’ social risks.

**Objective:**

This study’s aims are to develop electronic health record (EHR)–based clinical decision support (CDS) tools that suggest social risk–informed care plan adaptations for patients with diabetes or hypertension, assess tool adoption and its impact on selected clinical quality measures in community health centers, and examine perceptions of tool usability and impact on care quality.

**Methods:**

A systematic scoping review and several stakeholder activities will be conducted to inform development of the CDS tools. The tools will be pilot-tested to obtain user input, and their content and form will be revised based on this input. A randomized quasi-experimental design will then be used to assess the impact of the revised tools. Eligible clinics will be randomized to a control group or potential intervention group; clinics will be recruited from the potential intervention group in random order until 6 are enrolled in the study. Intervention clinics will have access to the CDS tools in their EHR, will receive minimal implementation support, and will be followed for 18 months to evaluate tool adoption and the impact of tool use on patient blood pressure and glucose control.

**Results:**

This study was funded in January 2020 by the National Institute on Minority Health and Health Disparities of the National Institutes of Health. Formative activities will take place from April 2020 to July 2021, the CDS tools will be developed between May 2021 and November 2022, the pilot study will be conducted from August 2021 to July 2022, and the main trial will occur from December 2022 to May 2024. Study data will be analyzed, and the results will be disseminated in 2024.

**Conclusions:**

Patients’ social risk information must be presented to care teams in a way that facilitates social risk–informed care. To our knowledge, this study is the first to develop and test EHR-embedded CDS tools designed to support the provision of social risk–informed care. The study results will add a needed understanding of how to use social risk data to improve health outcomes and reduce disparities.

**International Registered Report Identifier (IRRID):**

PRR1-10.2196/31733

## Introduction

### Background

The conditions in which people live, work, and play—known as social determinants of health (SDH)—have well-documented impacts on health care access and quality and also on health outcomes [1-3]. SDH are shaped by broader social, economic, and structural forces and contribute to long-standing, avoidable health disparities and inequities [2]. Given the growing recognition of the impact of SDH on health, many health and health care professional organizations (eg, the American College of Physicians and the National Academy of Medicine) now recommend systematically screening for and documenting patients’ experiences of social adversity, including social risk factors related to food, transportation, and housing insecurity, in electronic health records (EHRs) [4-8]. As social risk data become more available in EHRs, it is important to understand how care teams can use this information to improve patient outcomes, which might reduce related health inequities.

A 2019 National Academies of Sciences, Engineering, and Medicine [9] report on integrating social and medical care describes a range of uses for reported social risk data in clinical settings. One such use is to provide or link patients with reported social risks to relevant social services, such as providing food resources to food-insecure patients or otherwise targeting social risks within the context of care delivery (*social risk–targeted care*). The National Academies of Sciences, Engineering, and Medicine report also describes a category of interventions that use social risk data to adapt care plans to account for a given patient’s social risks (*social risk–informed care*) [10]. Research on *social risk–targeted care* suggests that linking patients with social needs to specific social services can improve health outcomes [10-13]. Far less is known about the adoption and impact of social risk–informed care, although such care plan adaptations might improve health for individual patients and contribute to reducing disparities in care outcomes. For example, a social risk–informed care plan adaptation for a patient experiencing homelessness might involve avoiding refrigerated medications; for a patient with diabetes and food insecurity, it might include modifying insulin doses based on monthly food benefit schedules [14,15]. A series of studies in the Veterans Health Administration system found higher rates of positive clinical outcomes associated with social risk–informed care plan adaptations [16-20]. However, care that incorporates information about patients’ social context is not systematically incorporated into chronic disease management. Previous research found that social risk–informed care occurs only 15%-22% of the time in diverse care settings [21,22].

Social risk information must be presented to care teams in a manner that is useful to them and does not disrupt clinical workflows to encourage the systematic delivery of contextualized, social risk–informed care. Numerous studies have shown that clinical decision support (CDS) tools embedded in EHR systems can enhance care quality by providing clinical information to care teams along with suggestions on evidence-based actions relevant to a given patient’s care [23-29]. Such tools can include reminders about overdue screenings, summaries of a given patient’s health risks, and care recommendations per current guidelines. However, to our knowledge, the use and impact of EHR-based CDS to support the provision of contextualized, social risk–informed care has not been assessed.

### Objectives

This paper describes the protocol for a National Institutes of Health (NIH)-funded study (COHERE; Contextualized Care in Community Health Centers’ Electronic Health Records; R01MD014886) designed to develop and test CDS tools that present care team members with a given patient’s social risk information and both recommend and facilitate care plan adaptations based on those risks. This study will test the hypothesis that providing CDS that alerts care team members to patients’ known social risks and recommends relevant care plan adaptations will result in improved health outcomes. This study’s focus is on hypertension and diabetes control; however, the results will have implications for a wide range of morbidities.

## Methods

### Setting

The study will be conducted among community health centers (CHCs) that are members of OCHIN (not an acronym). OCHIN is a nonprofit health center–controlled network that hosts a single Epic EHR for >600 primary care CHCs located across the United States. As part of previous NIH-funded studies (1R18DK105463 and 1R18DK114701) and OCHIN’s ongoing CHC-centric EHR modifications, a suite of EHR tools was designed to enable documentation of social risk data and the provision of related referrals [23,30-33]. These tools were activated OCHIN-wide in June 2016; as of April 2021, >700,000 social risk screening results in >400,000 unique patients have been documented using these tools. The tools were adapted as their implementation was based on user input and to ensure alignment with the Epic EHR’s 2018 social determinants module. At present, the tools enable users to flag targeted patients for social risk screening, select from several screening tools (Protocol for Responding to and Assessing Patients’ Assets, Risks, and Experiences; the Centers for Medicaid/Medicare Services’ Accountable Health Communities screening tool; social risk questions from the Institute of Medicine [now the National Academy of Medicine]; or individual social risk domains) [34-37], enter patient-reported social risk data via several interfaces, and allow patients to self-enter data through the patient portal or at the point of care. The tools can also be used to document patients’ priorities related to social needs and interest in related referrals. In the trial described here, the CDS tools to be developed and tested will draw information on patient-reported social risks from data documented through these existing tools.

### Conceptual Guide

**Figure 1:**
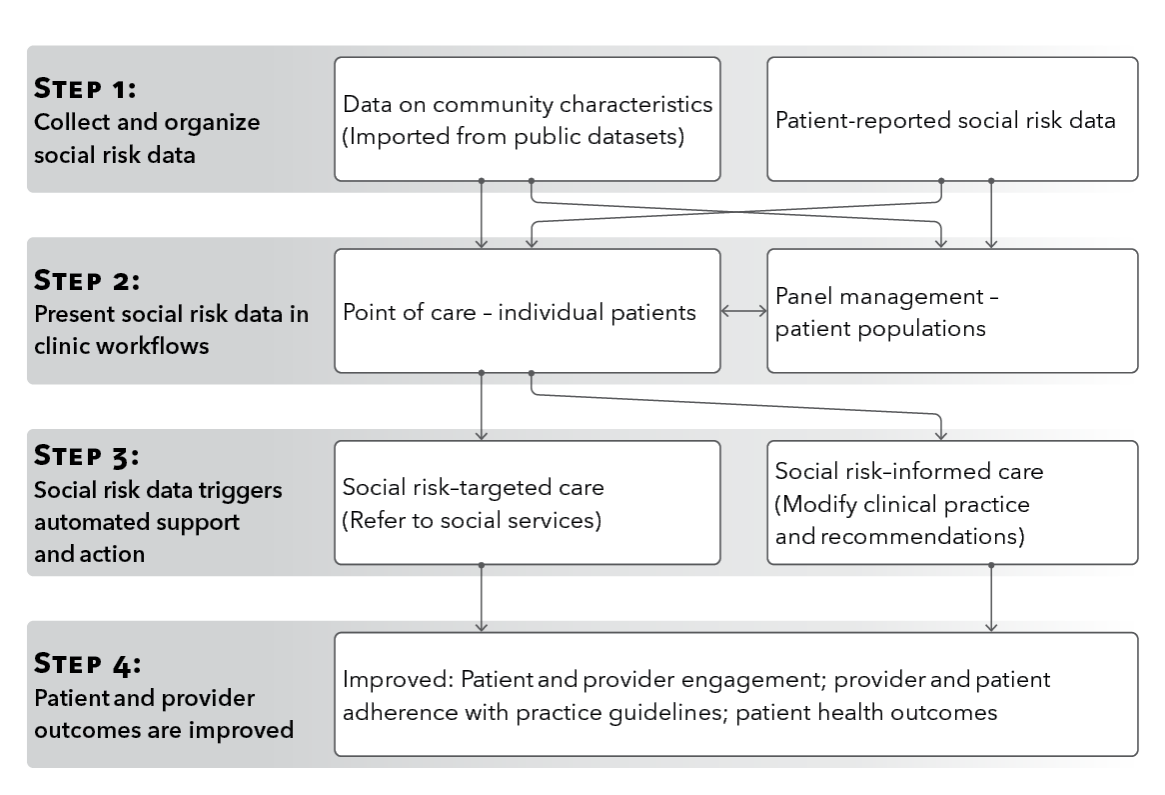
Social risk data and targeted or informed care (adapted from DeVoe et al [38]).

The conceptual framework proposed by DeVoe et al [38] for integrating SDH into primary care practice will guide the design of this intervention. A modified version of this framework (Figure 1) shows that data on self-reported social risks can be used to affect health care quality and outcomes either through panel management (eg, focused outreach) or at the point of care of individual patients through social risk–targeted care and social risk–informed care. This study focuses on EHR tools designed to facilitate point-of-care applications for social risk data, which have been proposed in theory but never formally tested.

### Study Design

A randomized quasi-experimental design will be used to assess the impact of the newly developed CDS tools designed to support social risk–informed care. Before beginning the main trial, several formative activities and a pilot study will be conducted.

#### Formative Phase

**Figure 2:**
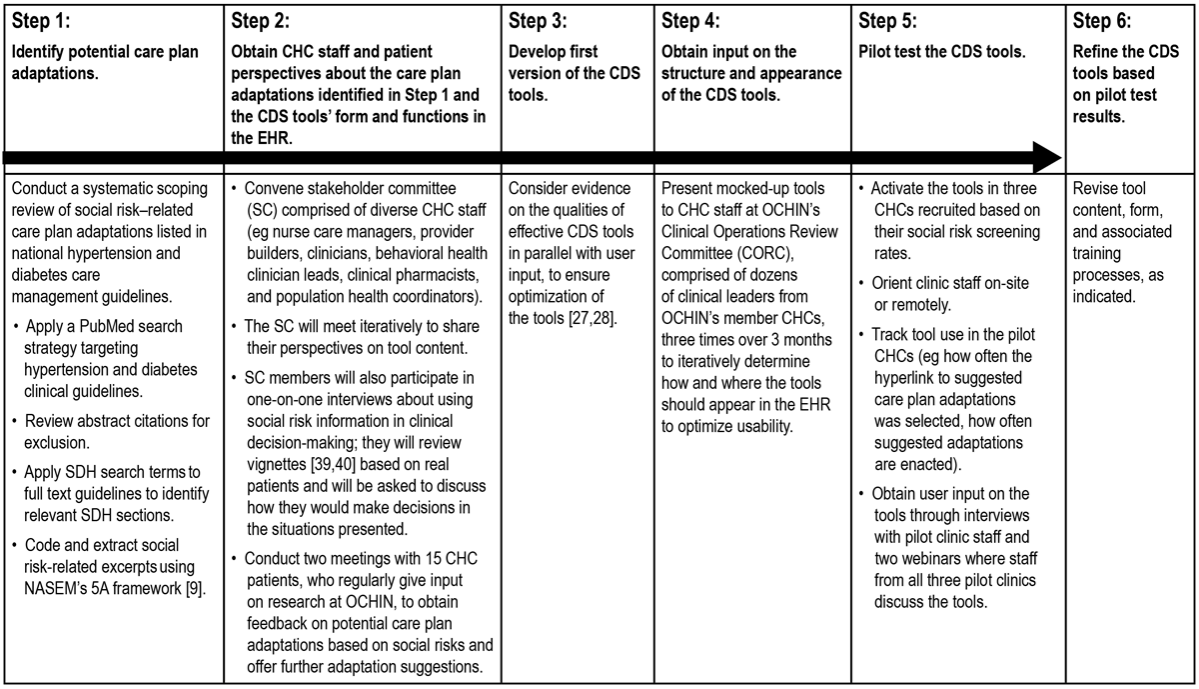
Steps involved in developing the clinical decision support tools. CDS: clinical decision support; CHC: community health center; EHR: electronic health record; NASEM: National Academies of Sciences, Engineering, and Medicine; SDH: social determinants of health [39,40].

First, potential care plan adaptations will be drawn from a systematic scoping review of social risk–informed care recommendations included in national hypertension and diabetes guidelines and our team’s prior research. Second, diverse CHC staff and patients will be asked to review and prioritize potential care adaptations. CHC staff will be invited to provide input through a stakeholder committee, whereas patients will be engaged using OCHIN’s established patient engagement panel. This process will help the research team determine (1) which social risks the study’s CDS tools will include, (2) the specific content of the CDS tools, and (3) the preferred form in which the CDS tools should appear in the EHR. The tools will then be pilot-tested for 12 months in three CHCs and further refined based on extensive user feedback from these pilot sites. Figure 2 outlines our process for developing the tools in preparation for the main trial.

#### Main Trial

Intervention clinics will have the CDS tools turned on in their EHR and will be followed for 18 months to assess (1) tool adoption, (2) the extent to which tool suggestions are enacted by care team members, and (3) the impact of tool use on two national clinical quality measures (CQMs) [41]: blood pressure control and hemoglobin A_1c_ (HbA_1c_) control. We will also assess care team members’ perceptions of the tools’ usability and impacts on care quality and patient-provider interactions.

### Randomization and Recruitment

All OCHIN clinics that provide primary care and have documented ≥200 social risk screenings (excluding the pilot clinics) will be identified. It is anticipated that approximately 60 clinics will meet these criteria. Eligible clinics will be randomized 1:1 to 1 of 2 groups: potential intervention or control. Clinics from the potential intervention group (n=30) will be recruited in random order until 6 agree to participate. In the study analyses, outcomes in these 6 intervention clinics will be compared with those in the control clinics (n=30). This approach allows for randomization between the intervention and control arms while eliminating the recruitment of CHCs to a study where some will receive no intervention (ie, only clinics that will receive the intervention will be contacted for recruitment). This method is possible as all OCHIN member CHCs agree that their EHR data may be used in research as part of their membership agreement.

### Intervention

Shortly before the tools are activated in participating CHCs, clinic staff members will be oriented to the CDS tools by an OCHIN EHR trainer (either on-site or remotely). Clinic staff will be offered a series of structured, sequenced activities using training materials developed by the study team in collaboration with the trainer. This will be the only form of implementation support offered to the main trial clinics as we seek to assess the adoption and impact of the CDS tools in a real-world situation.

### Analytic Framework

**Figure 3:**
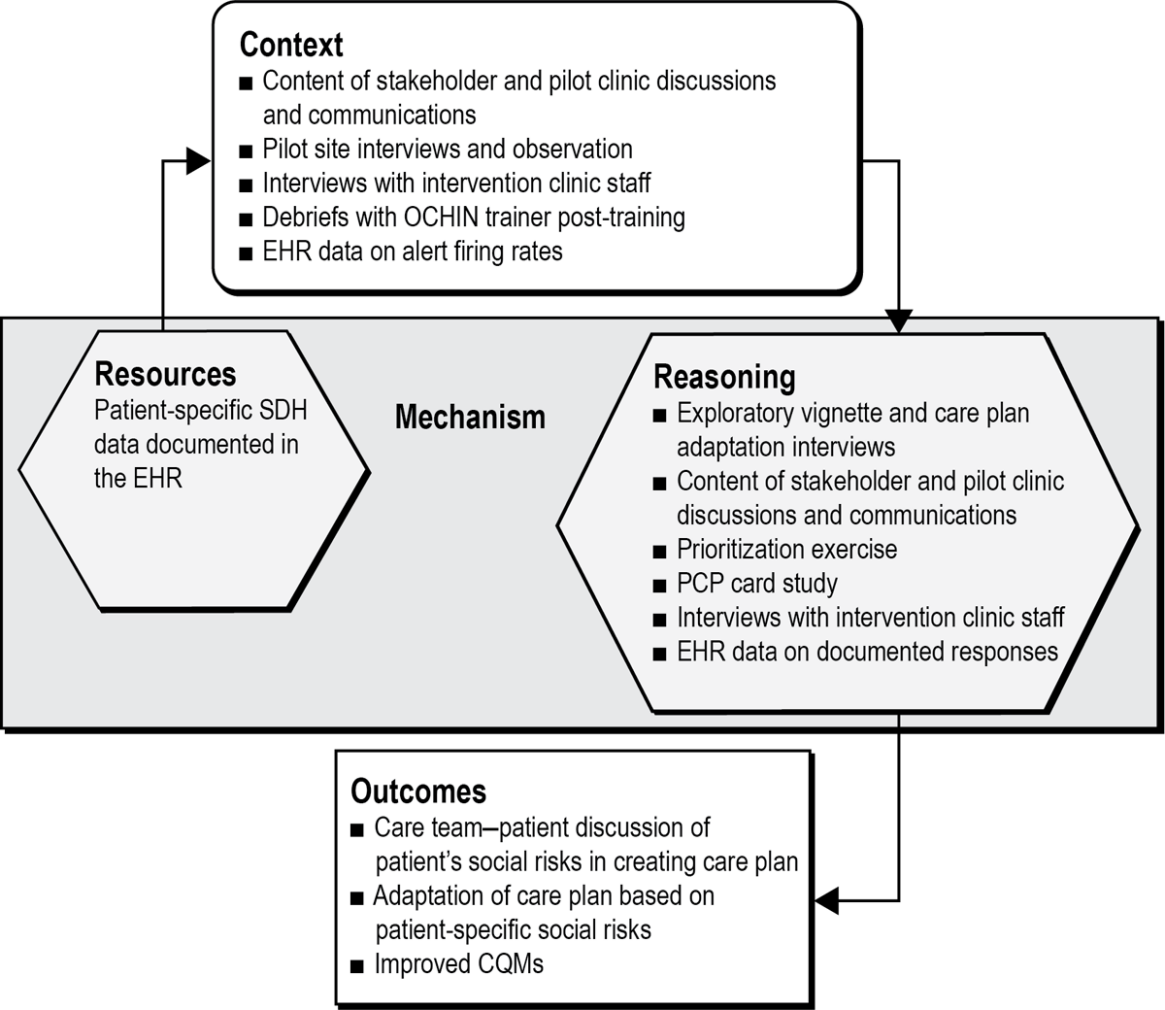
Realist evaluation framework. Resources: the resource or resources offered by the program under study. Context: pre-existing individual, social, institutional, economic and system-level values, norms, and relationships in which interventions are introduced. Reasoning: reasoning and reaction of stakeholders in response to resource or resources. Outcomes: intended and unintended changes resulting from the intervention. CHC: community health center; CQM: clinical quality measure; EHR: electronic health record; PCP: primary care provider; SDH: social determinants of health.

Mixed methods will be used to assess tool adoption and impact within a realist framework designed to identify what works, for whom, and in what circumstances [42,43]. The realist approach focuses on the context-dependent causal pathways through which an intervention (here, the CDS tool) produces outcomes (here, primarily adaptation of care plans based on patient-specific social risks and improved CQMs; Figure 3).

#### Data

All quantitative data will be extracted from OCHIN’s Epic EHR; these data are centrally managed and quality checked [44-46]. Qualitative data for the intervention phase will be collected via semistructured interviews with diverse clinic staff who interface with the CDS tools during patient care (eg, providers, care managers, and medical assistants) and an EHR-embedded provider card study at each study site. The interviews will explore relevant clinical contexts, perceptions and use of social risk data and CDS tools in clinical encounters, and the impact of patient-reported social risk data on clinical decision-making, quality of care, and patient-provider interactions, including potential negative impacts of tool use. Card studies are short (<1 minute) surveys designed to obtain point-of-care data on clinical decision-making [47]. This card study, which will be embedded within the EHR and, therefore, the provider workflow, will be used to assess provider reactions to and actions taken based on patient-reported social risk data.

#### Quantitative Analyses

We will describe the percentage of clinic encounters at which (1) the CDS tools appeared to users at the intervention clinics, (2) users reviewed tool suggestions, and (3) the suggested care plan adaptations were enacted.

The primary trial outcomes are changes in the two CQMs expected to be affected by social risk–informed care: blood pressure control and HbA_1c_ control. Each CQM’s denominator will be defined according to which patients are eligible for that measure at the time of a clinic visit (eg, patients with diabetes are in the HbA_1c_ measure denominator) per national CQM measurement specifications [41]. Two-level hierarchical linear models [48-50] will be used to assess the impact of CDS tools on these outcomes. As these outcomes are binary, the generalized form of the hierarchical linear model with a log link and binomial distribution will be used. Textbox 1 shows other potential covariates; this list will be finalized based on the extent to which person-level covariates were balanced between the final intervention and control arms and will be included in the first level of the model representing the person level. The second level of the model, the CHC level, will include arm as an independent variable (control vs intervention) and a random effect for the intercept (ie, intercept-as-outcomes model). Population-averaged marginal proportions and associated 95% CIs will be calculated by arm, along with the difference between those proportions (ie, the marginal effect) and the associated 95% CI that incorporates the random effects (ie, differences between CHCs) and any included covariates.

In secondary analyses, a repeated cross-sectional design will be used to assess the differential changes in the outcomes across time. A panel design is impractical in this population of CHC patients, so using repeated measures would result in a selective (eg, patients with stable and continuous care) and smaller sample. As we will be able to collect up to 18 months of data, we will subset the relevant patient subpopulations within clinics in 6-month increments and model time as a between-subjects effect in the model described above to evaluate the effectiveness for each outcome. More specifically, we will add time (6, 12, and 18 months) and the product of time and arm that represents their interaction in level 2 of the model. We will then calculate the population-averaged marginal proportions and associated 95% CIs by arm and time points, along with the marginal effects between arms within time points and between arms across pairs of time points (ie, differences-in-differences). Significant marginal effects between arms across time would suggest a differential change between the arms across time. As all secondary CQM outcomes (Textbox 1) are also binary, we will use the same approach to evaluate between-arm differences on these outcomes as for the primary outcomes.

Intent-to-treat analyses will be used to establish effectiveness at the population level and per-protocol analyses to assess the impact of the tools when used. For the primary analysis, depending on the size of the intraclass correlation (between 0.01 to 0.05), this study has at least 80% power to detect a 9%-17% difference in blood pressure control (n=343 per CHC; assuming baseline level of 60% blood pressure control calculated from OCHIN data) and a 10%-18% difference in HbA_1c_ control (n=214 per CHC; assuming baseline level of 34% HbA_1c_ control calculated from OCHIN data) between the intervention and control groups at a two-tailed α level of .05.

**Primary and secondary analysis variables.**

**Primary outcomes**

- Controlled blood pressure (<140/90)
- Controlled hemoglobin A_1c_ (in diabetes mellitus: <9%)

**Secondary outcomes**

- BMI ≥25 (adults; not a formal clinical quality measure: a health outcome associated with clinical quality measure)
- Controlled lipids (not a formal clinical quality measure: a health outcome associated with clinical quality measure)
- BMI screening and follow-up (adults)
- Lipid therapy (coronary artery disease)
- Use of aspirin or antithrombotic (ischemic vascular disease)

**Potential covariates**

- Patient covariates: Age, gender, race and ethnicity, primary language, poverty level, insurance status at a visit, and number of visits to that site or provider in last year (care use)
- Patient comorbidities: Charlson comorbidity score (modified)—an indicator of serious comorbid conditions that may shorten life expectancy
- Visit type:
  - In-person, telephone, and virtual
  - Outreach, encounter
- Provider type:
  - Degree (eg, medical doctor, registered nurse, or physician assistant)
  - Prescribing privileges (yes or no)
  - Number of patients in panel

#### Qualitative Analyses

Consistent with the constant comparative method, analyses will be iterative and inductive. Emergent understandings will be explored in subsequent data collection [51]. An immersion-crystallization process [52], which entails multiple iterations of data immersion, reflection, and code development and application, will be used to identify themes and patterns in the data [53].

#### Mixed Methods Realist Analyses

A convergent comparative *case analysis* approach will be used [54], where qualitative and quantitative data build an understanding of the change process in each *case* (clinic). Quantitative and qualitative data will be integrated, as shown in Figure 3. Data from each case will be *merged* for analysis and then compared within and across clinics to confirm, expand on, or challenge each site’s findings. Potential context-mechanism-outcome configurations [55-58] will be proposed and then refined as analysis continues to identify context-specific components that enable the use of patient-specific social risk data in adapting treatment plans. Emphasis will be placed on factors influencing the use of patient-specific social risk data in care decisions, including the use of CDS tools, any unintended negative impacts on care processes and outcomes, and patient-provider interactions. Analyses will also explore staff and patient perceptions of how patients’ social risk data affect care and the potential to induce or obviate bias when such actions occur.

## Results

This project was funded by the National Institute on Minority Health and Health Disparities of the NIH in January 2020. The formative phase started in April 2020 and will run through July 2021. The tool build process began in May 2021 and will continue through November 2022. Most of the tool development activities occurred during the first 3 months, with additional refinements occurring over subsequent months. The pilot study, which is part of the tool development period, will take place between August 2021 and July 2022. The main trial will begin in December 2022 and conclude in May 2024; qualitative interviews and a provider card study will be conducted during this time frame. Data analysis and dissemination activities will take place between December 2023 and November 2024.

The Kaiser Permanente Northwest institutional review board reviewed the study protocol and provided approval for pilot study activities in March 2021. The institutional review board will review the protocol again before the main trial. No research with human subjects will be conducted until the proper approvals have been received.

## Discussion

### Principal Findings

Although CHCs and other primary care providers are increasingly systematizing social risk documentation, their clinical teams lack guidance on how to use these data to improve patient health and reduce inequities [30,32,59-61]. Referring patients with social risks to needed social services is associated with improved health outcomes and decreased costs [10-13,21,62-80]. Although some of these impacts may occur because social service referrals reduce social risks, recent studies have shown that health improvements subsequent to social service referrals were not mediated solely by changes in social risk status [10,81]. Little is known about how to support social risk–informed care in CHCs or other primary care settings; however, research shows that when social risk data are presented without specific care recommendations, providers use these data inconsistently [22,82,83].

CDS tools in EHRs (eg, alerts, overviews, and care gap summaries) are widely available and can enhance care quality and patient satisfaction, especially when developed with user input [24,25,27-29,84-91]. These functionalities might enhance social risk–informed care; however, we know of no prior studies examining their use in this context. This trial will address this knowledge gap by developing and testing CDS tools that suggest social risk–informed care adjustments. The CDS tools that will be tested will be developed and revised with extensive provider and patient stakeholder inputs. These tools will be designed to enable transferability to any site using the Epic EHR; general principles of using social risk data CDS for social risk–informed care will be disseminated so that similar tools can be built in any EHR system.

### Limitations and Considerations

This pragmatic trial will be conducted in CHC settings as CHCs serve patients with high rates of social risks, so study findings may not be fully generalizable to other care settings. Although the recruited CHCs will have documented social risks for ≥200 patients, few will have screened all their patients for social risks, and some will not have endorsed social risks. As a result, these tools may not be relevant to all patients. For addressing this, primary analyses will be limited to patients with known social risks, and differences between these patients and those with no social risk data will be described. Only 1-2 hours of training will be provided as implementation support to the study CHCs; more hours of training might be optimal but would not reflect real-world practices. Finally, this research was conducted in the peri-COVID period; its generalizability must be interpreted in light of the pandemic’s effects on patients’ health care access and financial security, as well as the changes it spurred in the health care sector around social care delivery [92]. The extent to which pandemic-related changes will endure is not yet clear.

### Conclusions

There are no known prior studies assessing whether and how EHR-based CDS can be used to support contextualized social risk–informed care. We believe this study will be the first to develop and test CDS tools that both highlight CHC patients’ social risks and suggest care plan adaptations based on reported social context. The tools will be developed with extensive input from CHC staff and patients to ensure patient and provider usability and acceptability. The results will yield usable data for CHCs and other primary care providers to inform strategies that can ensure new social risk screening initiatives translate to improvements in care delivery and health outcomes.

## Abbreviations

CDS: clinical decision support
CHC: community health center
COHERE: Contextualized Care in Community Health Centers’ Electronic Health Records
CQM: clinical quality measure
EHR: electronic health record
HbA_1c_: hemoglobin A_1c_
NIH: National Institutes of Health
SDH: social determinants of health
